# RNAseq Analysis of Rhizomania-Infected Sugar Beet Provides the First Genome Sequence of Beet Necrotic Yellow Vein Virus from the USA and Identifies a Novel Alphanecrovirus and Putative Satellite Viruses

**DOI:** 10.3390/v12060626

**Published:** 2020-06-10

**Authors:** John J. Weiland, Roshan Sharma Poudel, Alyssa Flobinus, David E. Cook, Gary A. Secor, Melvin D. Bolton

**Affiliations:** 1 United States Department of Agriculture, Agricultural Research Service, Northern Crop Science Laboratory, Fargo, ND 58102, USA; John.Weiland@usda.gov (J.J.W.); alyssa.flobinus@ndsu.edu (A.F.); 2 Department of Plant Pathology, North Dakota State University, Fargo, ND 58108, USA; roshan.sharmapoudel@ndsu.edu (R.S.P.); Gary.Secor@ndsu.edu (G.A.S.); 3 Department of Plant Pathology, Kansas State University, Manhattan, KS 66506, USA; decook@ksu.edu

**Keywords:** sugar beet, rhizomania, RNAseq, virus, necrovirus, helper virus

## Abstract

“Rhizomania” of sugar beet is a soilborne disease complex comprised of beet necrotic yellow vein virus (BNYVV) and its plasmodiophorid vector, *Polymyxa betae*. Although BNYVV is considered the causal agent of rhizomania, additional viruses frequently accompany BNYVV in diseased roots. In an effort to better understand the virus cohort present in sugar beet roots exhibiting rhizomania disease symptoms, five independent RNA samples prepared from diseased beet seedlings reared in a greenhouse or from field-grown adult sugar beet plants and enriched for virus particles were subjected to RNAseq. In all but a healthy control sample, the technique was successful at identifying BNYVV and provided sequence reads of sufficient quantity and overlap to assemble > 98% of the published genome of the virus. Utilizing the derived consensus sequence of BNYVV, infectious RNA was produced from cDNA clones of RNAs 1 and 2. The approach also enabled the detection of beet soilborne mosaic virus (BSBMV), beet soilborne virus (BSBV), beet black scorch virus (BBSV), and beet virus Q (BVQ), with near-complete genome assembly afforded to BSBMV and BBSV. In one field sample, a novel virus sequence of 3682 nt was assembled with significant sequence similarity and open reading frame (ORF) organization to members within the subgenus *Alphanecrovirus* (genus *Necrovirus*; family *Tombusviridae*). Construction of a DNA clone based on this sequence led to the production of the novel RNA genome in vitro that was capable of inducing local lesion formation on leaves of *Chenopodium quinoa*. Additionally, two previously unreported satellite viruses were revealed in the study; one possessing weak similarity to satellite maize white line mosaic virus and a second possessing moderate similarity to satellite tobacco necrosis virus C. Taken together, the approach provides an efficient pipeline to characterize variation in the BNYVV genome and to document the presence of other viruses potentially associated with disease severity or the ability to overcome resistance genes used for sugar beet rhizomania disease management.

## 1. Introduction

The increasing globalization of food and other commodities has resulted in greater exposure of crops to historical and emerging pests and diseases, including viruses [1,2]. Within a pathosystem, novel pathogen variants acting alone or in combination may arise to compromise existing resistance in the crop [3,4,5]. Because of the unanticipated complexities wrought by such phenomena in disease development and crop protection, accurate, rapid and, increasingly, naïve diagnostics and analyses are required to keep pace with the accelerating diversity in pests and pathogens.

Sugar beet contributes nearly half of the sucrose produced and consumed within the United States of America (USA) [6]. “Rhizomania” is considered to be the most devastating root disease of sugar beet globally and is managed principally through genetic resistance in the host [7,8]. The disease is caused by the beet necrotic yellow vein virus (BNYVV; family *Benyviridae*, genus *Benyvirus*). Like most plant viruses, BNYVV is a positive-sense RNA virus. The genome of BNYVV is divided among 4 or 5 segments (designated RNAs 1 to 5), the roles of which, during infection, have been the source of intense investigation over the past 30 years [9,10,11,12]. Thus, genes, as well as non-coding regions, have been assigned functions in virus replication, encapsidation, intra- as well as inter-plant movement, silencing suppression, and pathogen aggressiveness [10,13]. Recent studies on the latter of these functions have led to the discovery of a highly mutable region in the *p25* gene on RNA 3, implicated in the breaking of monogenic resistance provided by the Rz1 gene [5,14,15,16,17]. The Rz1 gene was discovered in sugar beet germplasm in the 1980s and, even with the breaking of this resistance by strains of BNYVV worldwide, it remains the most widely used gene for the management of this disease [6]. Nevertheless, many questions remain regarding the full nature of resistance-breaking by BNYVV in sugar beet, the extent to which the plasmodiophorid vector of BNYVV, *Polymyxa betae*, plays a role in disease aggressiveness, and the degree to which other known, and possibly unknown, viruses in the sugar beet root infection court positively or negatively impact disease.

Rapid and accurate genetic analysis of known and potential pathogens is a fundamental goal of disease management programs as the genetic constitution of a pathogen population may be crucial in determining their disease-causing potential. The advent of “next-generation sequencing” (NGS) technologies for DNA and RNA analysis, combined with the increasing power of computational platforms, has removed a long-standing roadblock in rapid, cost-effective, population-scale sequence generation and genome examination [18,19]. In cases where RNA transcripts or RNA viruses are the targets of an investigation, RNAseq has become a widely used approach to obtain sequence representation of all RNA in a sample, with sample fractionation offering a way to bias the RNA population toward desired targets [20,21]. An added power to RNAseq is that it is “naïve”, remaining relatively unbiased with respect to the molecules contributing to the final sequence assembly. Thus, RNAseq offers a powerful tool for generating sequenced transcriptomes from multi- and unicellular organisms and for producing RNA “viromes”–the global representation within a sample of all RNA viruses present [22].

In the present study, samples from diseased sugar beet seedlings and adult plants were enriched for viruses and subjected to RNAseq analyses. Reads were assembled to produce genomic segments, or in some cases, open reading frames (ORFs), which were used to identify potential relatedness of the viral genomes obtained to existing viral sequences in public databases. Based on the derived BNYVV sequence of RNAs 1 and 2 and that of a previously uncharacterized necrovirus, infectious cDNA clones were developed, validating the integrity of the approach. With the additional discovery of potentially novel satellite viruses, the results confirm the usefulness of the method in assessing the spectrum of viruses present in sugar beet plants exhibiting rhizomania disease.

## 2. Materials and Methods

### 2.1. Sample Collection

Three soil samples (S1–S3) and one root sample (S6) were obtained from the sugar beet production areas of the Red River Valley of North Dakota and Minnesota and southern Minnesota by agriculturalists of the Min-Dak Farmers Cooperative (Wahpeton, ND, USA) and Southern Minnesota Beet Sugar Cooperative (Renville, MN, USA) in 2018 (Table S1). Two soil samples (S4 and S5) were also received from sugar beet production areas in Idaho by agriculturalists of The Amalgamated Sugar Co. (Boise, ID, USA) in 2017 (Table S1).

### 2.2. Plant Growth and Virus Recovery

To recover BNYVV from infested soil samples, we followed the methodology described by Weiland et al. [17]. Briefly, rhizomania-susceptible sugar beet seeds of the cultivar SLC4-K583-G1C (SES VanderHave; Tienen, Belgium) were sown in 250 mL pots containing one-part infested soil to one-part sterile sand. As a negative control, seeds were planted into sterile sand mixed 1:1 with Sunshine Mix #1 (Sungro Horticulture; Agawam, MA, USA). Slow-release fertilizer (Multicote; Sungro Horticulture; Agawam, MA, USA) was added following the manufacturer’s instructions. Plants were grown in a greenhouse under standardized conditions at 24 °C (day)/18 °C (night), with 8 h of supplemental light per day. Water was added directly as needed. Six weeks after planting in infested soil, plants were harvested, and a root sample consisting of 5 to 7 seedlings was taken from each pot. Roots were washed under running tap water, tamped dry on paper toweling, and stored on ice in plastic bags in preparation for virus extraction. Freezing of samples was avoided, which can compromise the integrity of the viral RNA upon sample thawing. ELISA to detect BNYVV was performed on parallel samples in order to determine the best replicates to use for virus extraction. For Sample 6, comprised of single mature, field-grown sugar beet root, the hairy roots characteristic of the disease were washed thoroughly in tap water, excised from the root surface using care to include a portion of the necrotic veinal tissue in the sample and processed for virus enrichment as described below.

### 2.3. Virus Enrichment

Efforts were made to enrich for virus particles using standard PEG precipitation of crude extracts [23]. Briefly, fresh root tissue was ground with a mortar and pestle in cold 0.1 M NaPO_4_ pH 5.2 (3 mL buffer per g fresh weight of tissue). Each homogenized sample was transferred as 1.0 mL aliquots to several 1.5 mL microcentrifuge tubes, each containing 0.4 mL of chloroform, and the contents mixed well and then centrifuged at 16,000× *g* at 4 °C for 18 min. Supernatants were transferred to new tubes, adjusted to 1% NaCl and 8% PEG 8000, and incubated on ice for 10 min. Following a centrifugation step for 18 min at 16,000× *g* at 4 °C, the supernatant was removed and discarded. The whitish pellet was resuspended in 0.4 mL of cold 0.1 M NaPO_4_ pH 5.2 and the sample extracted with 0.1 mL of chloroform. The aqueous phase from the extraction was precipitated once again by the addition of NaCl to 1% and PEG 8000 to 8%, and samples were incubated on ice overnight. The following day, the samples were centrifuged at 4 °C for 18 min at 16,000× *g*. Care was taken to remove to the extent possible all PEG supernatant from the small pellet. RNA was extracted from the pellet, as previously described in Weiland and Edwards [24]. The integrity of the recovered RNA was determined using agarose gel electrophoresis, and the RNA quantified using spectrophotometry. Shipment of samples to commercial producers of RNAseq data followed the instructions of the contractor.

### 2.4. RNAseq Analyses

RNAseq libraries (150 bp insert size) were prepared and sequenced (pair-end 100 bp) by BGI Americas (Cambridge, MA, USA) or Admera (South Plainfield, NJ, USA) using the Illumina Highseq 4000 sequencing platform. Customized bioinformatics was also provided by each company. Briefly, low-quality reads and adaptor sequences were removed. For each sample, short reads were de novo assembled with different k-mer sizes in parallel. Reads were subsequently mapped back to the assembled contigs for validation. The best assembly was chosen based on contig N50 and mapping rate. Standard BLAST queries were used to verify or postulate the identification of novel viruses discovered in this work and for confirming the accuracy and completeness of genome assemblies.

To identify known sugar beet viruses, high-quality filtered reads from each sample were mapped to reference genome sequences of BNYVV (GeneBank assembly accession: GCF_000854885.1), BSBMV (GeneBank assembly accession: GCA_002867265.1) and BBSV (GeneBank assembly accession: GCF_000855285.1). Mapping was done using default parameters (except length fraction = 0.8) in CLC genomics workbench v 8.0 (CLC Bio, Qiagen, Germantown, MD, USA). Any reads (paired- and single-end reads) mapped to a given viral genome were extracted for de novo assembly in CLC genomics using default parameters (word size = automatics; bubble size = automatic). A near-complete genome assembly of BNYVV, BSBMV, and BSBV was obtained from the majority of the samples. Assembly of sequences from Sample 3 also suggested the presence of a novel virus with sequence characteristics of plant Alphanecroviruses and potential satellite viruses.

### 2.5. Sequence Analysis of BNYVV Strains

The de novo generated BNYVV RNA sequences were BLASTn (https://blast.ncbi.nlm.nih.gov) searched against publicly available BNYVV sequences to inspect the percentage identity (homology) between them. Nucleic acid consensus sequences for BNYVV isolates collected as part of this work were designated according to sample number. Assembled sequences of RNAs 1 to 4 of BNYVV were used to infer ORF locations. The analysis of sequence relatedness between RNAs 1 and 2 from this study (GenBank accessions MT227164 and MT227165, respectively) and those in GenBank was performed using BLAST. Due to the larger number and wider geographic distribution of available sequences for BNYVV RNAs 3 and 4, genes *p25* and *p31* (from RNA3 and RNA4, respectively; GenBank accessions MT372831-MT372842) were analyzed. Multiple sequence alignment of *p25* sequences from 52 strains and for *p31* from 46 strains were carried out using MUSCLE v3.8.31 (https://www.ncbi.nlm.nih.gov/pmc/articles/PMC390337/). The phylogenetic relationships were inferred using Randomized Axelerated Maximum Likelihood (RAxML) v8.2.9 (https://www.ncbi.nlm.nih.gov/pmc/articles/PMC3998144/). Our RAxML analysis utilized rapid bootstrap analysis to search for the best-scoring ML tree with the number of bootstrap iterations determined at runtime using the extended majority-rule consensus tree criterion (i.e., bootstopping), and the “GTRGAMMA” model of nucleotide substitutions. The best-scoring ML tree for each gene was visualized in FigTree v1.4.4 (https://github.com/rambaut/figtree). Orthologous genes from BSBMV were tested for inclusion in the analysis to serve as an outgroup, but the sequences were too divergent, a similar conclusion as previously reported [25]. As such, we employed midpoint rooting in FigTree.

### 2.6. Construction and Inoculation of BNYVV RNA1 and 2 Infectious Clones

Based on the data obtained in the present work from RNAseq and conserved sequences at the 5′- and 3′-termini in BNYVV genomes from across the globe (Table 2), clones of RNA 1 and 2 were designed for construction. For BNYVV RNA 1 cDNA, four synthetic overlapping fragments were generated by Genewiz (South Plainfield, NJ, USA) and delivered as discrete fragments in vector pUC57, which were used as a template to produce four PCR amplicons (primer sequences in Table S2). *BamH*I-T7 promoter (25 nt) and polyA_60_-*Hind*III-*BamH*I (72 nt) sequences were incorporated on the 5′ end of the first fragment and the 3′ end of the fourth fragment, respectively, by PCR. Subsequently, these amplicons were used as templates in overlap extension PCR (Table S2) to generate the full-length cDNA of RNA 1. The entire sequence of the RNA1 cDNA (6747 nt genome) was cloned into the pNEB193 vector (New England Biolabs, Waltham, MA, USA) using *BamH*I restrictions sites. The full-length BNYVV RNA 2 cDNA clone (4610 nt genome) was synthesized by Genewiz and delivered in a pSMART-BAC (Lucigen Inc., Middleton, WI, USA) vector flanked by two *Bam*HI restriction sites. The RNA 1 and RNA 2 clones were linearized by *Bam*HI and *Hind*III restriction enzymes, respectively, and used to produce capped, polyadenlylated RNA using methods previously described [24]. Inoculation of the transcript RNA to leaves of *Chenopodium quinoa* followed the procedure of Petty et al. [26], and Western blotting and detection of BNYVV-infected leaves using an anti-BNYVV antibody (Agdia Inc., Elkhart, IN, USA) were performed according to Weiland and Edwards [24].

### 2.7. Putative Alphanecrovirus and Satellite Virus Sequence Validation and Characterization

Primers for cDNA synthesis and DNA amplification and sequencing were designed based upon sequences produced through RNAseq and from sequence accessions in public sequence databases. Reverse transcription and polymerase chain reaction (RT-PCR) conditions, using primers indicated below, were as outlined in Edwards et al. [27]. Amplification of putative satellite sequences encoding the predicted coat protein was done using primers MDB-1867 and MDB-1868 (Table S2). Primers MDB-2100 and MDB-2101 (Table S2) were employed to generate a single amplicon from the putative novel Alphanecrovirus in sample S3. The amplicon sequence originates in the 3′-end of the p52 ORF, spans the p8 and p6 ORFs, and encompasses the entire predicted P30 CP gene (Figure 4). The sequence of the P23 ORF subsequently was found within the raw sequence reads extending the assembled sequence towards the 5′-end of the genome. Finally, for both the novel Alphanecrovirus and the satellite virus, the SMART RACE kit (TaKaRa Bio Inc., Mountain View, CA, USA) was employed to capture and characterize 5′- and 3′-end sequences.

### 2.8. Construction and Inoculation of Novel Alphanecrovirus Infectious Clones

Full-length clone construction for the novel Alphanecrovirus was initiated by generation of a genome-length amplicon using primers MDB-2460 and MDB-2462 (Table S2) in which the first 17 nt of MDB-2460 comprise the phage T7 RNA polymerase promoter. The amplicon was blunt-cloned into pMiniT 2.0 (New England Biolabs, Waltham, MA, USA). Two clones (pBvANV#7 and pBvANV#10) were linearized with *Eco* R1 restriction enzyme and transcribed in vitro, as described previously, for the generation of infectious RNA of the Betanecrovirus BBSV [28]. Inoculation of expanded leaves of healthy *C. quinoa* with the synthetic RNA derived from clones pBvANV #7 and 10 also followed the methods of Weiland et al. [28]. ELISA analysis of protein extracts prepared from diseased and healthy *C. quinoa* leaves employed the same methods as noted above for the detection of BNYVV CP but using a TNV-A detection kit (Loewe Diagnostics, Sauerlach, Germany).

## 3. Results

### 3.1. Virus Enrichment and RNAseq Analysis

**Figure 1:**
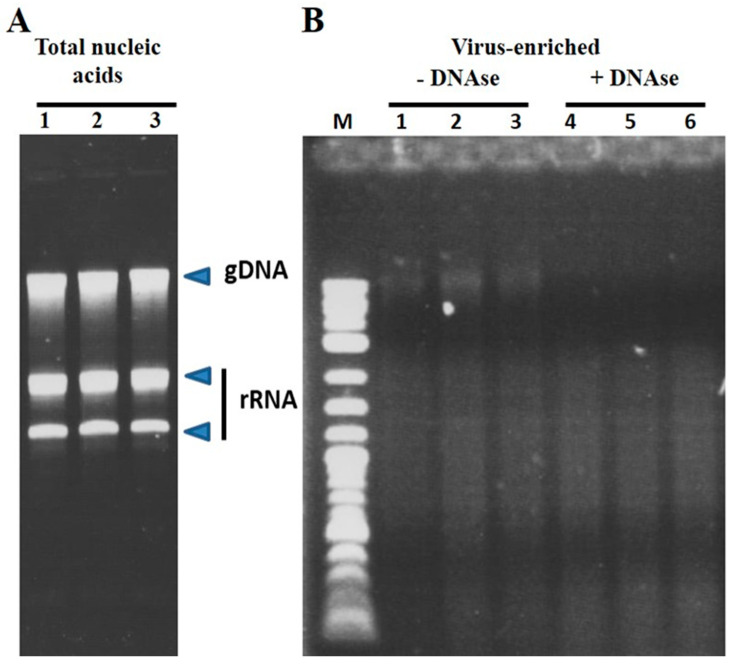
Agarose gel electrophoresis comparing total RNA prepared from sugar beet roots versus using virus-enriched RNA. (**A**) Typical preparation of total nucleic acids from roots of 6-week-old sugar beet plants is dominated by genomic DNA and ribosomal RNA. (**B**) Virus-enrichment removes the bulk of the cellular RNA, although additional DNase treatment (compare -DNase and +DNase lanes) is required to remove residual genomic DNA in preparing the sample for RNAseq. A DNA size standard (M) is included to monitor the approximate size of the RNA population.

The use of polyethylene glycol in virus precipitation has been employed in virus purification for decades [23] and provided the basis for the enrichment in the present study. An example of the efficacy in the enrichment of viral RNA over cellular nucleic acids is shown in Figure 1 and Figure 5, where removal of the bulk of rRNA and genomic DNA is evident. Additionally, this is also evident in Figure 5, where a satellite virus genome is clearly the most abundant product of the enrichment scheme. Approximately 20 to 40 ng of prepared RNA sufficed to produce libraries for each sample capable of yielding reads for the assembly of multiple virus genomes present in the samples.

Approximately 60,000,000 RNAseq reads were obtained per sample (Table 1). Using BLAST alignments to assign reads to known virus sequences in the NCBI database, numerous confirmed (Table 1) and potential sugar beet viruses were detected within the samples (see Figure S1 for maps of main viruses observed). The viruses BNYVV, BSBMV [29], and BBSV [28] are known to produce disease symptoms on infected sugar beet plants, whereas plants infected by BSBV [30] and BVQ [31] are relatively asymptomatic. BBSV was first reported in the USA in 2006 in Colorado [32], the present work documenting a more western distribution of this virus in the USA than previously known (near-complete and partial genome sequence found in Samples 4 and 5, respectively, Gooding County, ID, USA). The proportion of the genome able to be assembled for each of the viruses varied between viruses within a sample but was over 92% for all BNYVV RNAs (Table 1). The viruses BNYVV and BSBMV were generally associated with the highest proportion of viral sequence reads. This is consistent with the fact that the study targeted samples with the highest probability of inducing Rhizomania disease. The genome sequence of BSBMV was nearly identical to that reported by Lee et al. [33], with less than 0.01% nucleotide differences observed. Sample S6 differed from all other samples in being obtained from field-grown sugar beets in southern Minnesota. The virus complement was not markedly different from those obtained through baiting of the viruses from soil in a greenhouse setting, even as the number of reads of BNYVV predominated in the sample.

### 3.2. Taxonomic Grouping of US BNYVV Isolates and Biological Validation of Sequences

**Figure 2:**
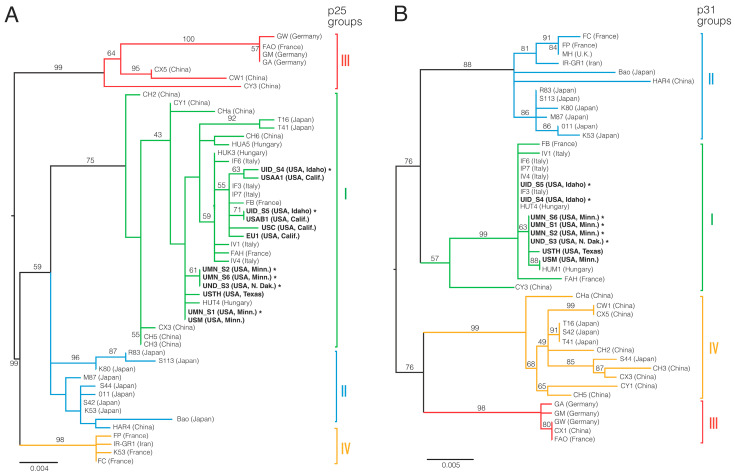
Relatedness of USA BNYVV to global isolates based on *p25* and *p31* gene sequences. Phylogenetic trees were inferred using Randomized Axelerated Maximum Likelihood (RAxML), using runtime calculated bootstrapping. (**A**) Coding sequence of p25 from 52 BNYVV strains and (**B**) coding sequence of p31 from 46 strains, coded on RNA3 and RNA4 of the BNYVV genome, respectively. Each tree was midpoint rooted, and ML support values are displayed for each branch with greater than 50% support. The four resulting groups in each tree were colored and indicated with solid bars shown to the right of each tree. The strain name is shown for each isolate along with the reported country of isolation in parentheses along with the state for US isolates. All US isolates are in bold. Sequences collected from this study are denoted with *.

Analysis of the BNYVV genomes obtained in the study indicated low-level variation in the sequence of RNAs 3 and 4 from different sampling locations, but no significant variation between sampling sites for RNAs 1 and 2. The most variable region of the BNYVV genome worldwide is the noted “tetrad region” encoded on RNA3 between nucleotides 645 and 656 (E12 strain, NCBI Accession #EU330455.1; [5]). The most common tetrad amino acids (AAs) observed in the samples of the present study included VCHG, ACHG, VLHG, and TLHG, the first three of which have a prior association with the breaking of Rz1-gene-based resistance in sugar beet [5,14,17]. All sequences were used to confirm the placement of the BNYVV-US within phylogenetic groupings (Figure 2) previously reported by Chiba et al. [25], Schirmer et al. [34], and Zhuo et al. [35]. Through the ranking of similarity with previous sequences already used in phylogenetic group formation (Table 2; Figure 2), the lineage of the US isolates obtained based on sequence analysis was in broad agreement with the results of these prior analyses, indicating that these viruses are of the A-type designation. Additionally, the sample sequences provided evidence of the continued absence of RNA-5 from the US, as has been noted previously [6].

### 3.3. Development of Infectious Clones of BNYVV RNAs 1 and 2

**Figure 3:**
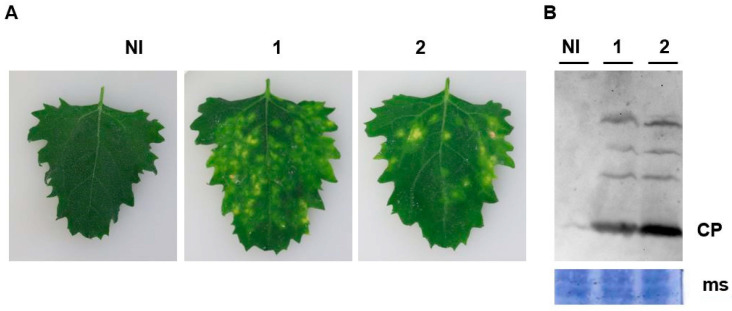
Capped transcripts RNAs 1 and 2 produced from USA BNYVV. (**A**) cDNA clone constructs are infectious in *Chenopodium quinoa*. Transcripts RNAs 1 + 2 combined and rub-inoculated to *C. quinoa* leaves induced chlorotic local lesions from 5 days postinoculation (panels 1–2). (**B**) Total proteins were extracted from local lesions and viral coat protein (CP) was detected by Western blot using anti-BNYVV antiserum (lanes 1–2). Membrane staining (MS) with Coomassie brilliant blue to provide a loading standard. Noninoculated (NI) plants were used as controls. 1 and 2 represent two plants inoculated with RNAs 1 + 2, respectively.

Given that RNAseq produces a consensus sequence from an RNA virus population that may exhibit underlying variation and that RNAs 1 and 2, nevertheless, were highly invariant across samples, we reasoned that DNA clones synthesized based on the obtained sequence would have a high probability of being infectious. However, the obtained sequences did not include the terminal nucleotides represented in previously confirmed sequences of BNYVV (Table 1). Alignment of RNA 1 and 2 sequences presented in Table 2 confirmed that the terminal sequences appeared invariant in BNYVV across continents. Consequently, we incorporated these nucleotides not present in the RNAseq data in the terminal primer sequences towards the production of infectious BNYVV RNA 1 and RNA 2 clones. With the historical difficulty in producing and maintaining clones of RNA 1 and RNA 2 in multiple laboratories across the globe, we elected to construct these in a BAC vector using a commercial gene synthesis service. Success in this approach was achieved with RNA 2, but RNA 1 proved intractable for cloning by this method. Instead, an RNA 1 clone was obtained within our research laboratory in which the insert cDNA copy was (a) produced as a full-length 6.7 kb amplicon from source materials, (b) identical in sequence to that submitted to the commercial service for synthesis, and (c) successfully maintained and amplified in pNEB193, a high-copy number plasmid vector. Linearization of both the RNA-1-possessing plasmid clone and the RNA-2-possessing BAC clone permitted the synthesis in vitro of capped transcripts representing both genome components. Infectivity of the capped, poly-adenylated transcripts produced abundant lesions on inoculated *Chenopodium quinoa* plants, the cause of which being due to BNYVV infection was confirmed by Western blot analysis (Figure 3). A more complete description of the clones and their use in BNYVV variant analysis is in progress in our laboratory (Flobinus et al., in preparation).

### 3.4. Novel Virus Discovery in Sugar Beet through RNAseq

The RNA prepared from the enriched virus fraction of Sample S3 possessed an abundant RNA species of ~1.2 kb, an unanticipated outcome of the project. Subsequently, standard total RNA extraction from infected plants and quality analysis by agarose gel electrophoresis revealed the RNA to be visible in agarose gel analysis even without virus particle enrichment (Figure 5). Application of RNAseq to the enriched Sample S3 resulted in greater than 50% of the total reads being assigned to this RNA. Agarose gel electrophoresis of the 1.2 kb RNA suggested that it was a mixture of two isoforms, an observation corroborated by RNAseq reads that were able to be assembled into two related variants (designated BvSatVirus1A and BvSatVirus1B; GenBank accessions MT227166 and MT227167, respectively; Figure S2). A single major ORF present on both RNA isoforms encoded a predicted amino acid sequence with weak similarity to satellite maize white line mosaic virus (MWLMV) [36], a result consistent with the apparent size of the RNA and the recovery of the RNA via virus enrichment.

**Figure 4:**
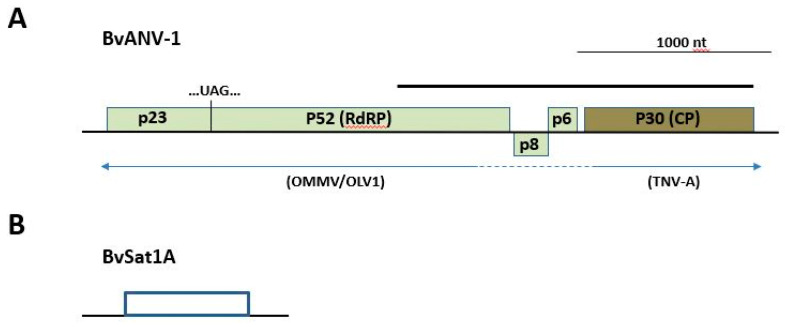
Genome map approximating the organization and size of putative novel viruses associated with sugar beet. (**A**) In the novel *Alphanecrovirus* (BvANV-1), the 5′-proximal ORF is interrupted by an amber stop codon (UAG), as is characteristic for several members of the *Tombusviridae,* and its predicted protein product possesses, along with the predicted movement proteins p8 and p6, greatest similarity to those of the closely related viruses OLV-1 and OMMV. The predicted coat protein (P30-CP) is more closely related to that of TNV-A. Both the 5′- and 3′-UTRs possess the greatest similarity to those from TNV-A. The solid bar above the map represents the single amplicon produced within the study, validating the integrity of the discovered viral genome. (**B**) Approximate genome size and predicted coding information for satellite virus BvSat1A. A single ORF encodes a putative protein with similarity to satellite maize white line mosaic virus.

Additional reads within Sample S3 revealed the presence of the expected sugar beet viruses noted previously, as well as a potential variant of tobacco necrosis virus A (TNV-A; Meulewaeter et al. [37]; Figure 4). Detailed analysis revealed the putative virus sequence (GenBank accession MT227163) to be a potential hybrid between olive mild mosaic virus (OMMV; Cardoso et al. [38]) or olive latent virus-1 (OLV-1; [39]) from the 5′-half through the p8 and p6 genes and TNV-A (CP and 3′-UTR; Figure 4 and Table 3) for a total length of 3682 nt. Uncapped transcript RNA synthesized from a cloned copy of the sequence was demonstrated to be biologically active, producing necrotic local lesions on *C. quinoa*, characteristic of members of the *Alphanecrovirus* group (Figure S3). A more complete characterization of this putative OMMV/TNV-A hybrid and the satellites discovered in this work is in progress in our laboratory (Weiland et al., in preparation). Finally, Sample S4 additionally produced RNAseq reads, enabling the assembly of a variant of satellite tobacco necrosis virus C (98% query coverage possessing 81.4% nt identity with sTNV-C; Accession NC_043430.1).

## 4. Discussion

Since the advent of the polymerase chain reaction in the mid-1980s, applications of the technology have revolutionized the collection and analysis of DNA and RNA sequence information. RNAseq is one such application that combines high-throughput, short sequence reads of randomly-generated RT-PCR products with contemporary computational power, resulting in “shotgun” sequencing of the RNA population comprising a given sample [40]. Following on published successes using the method to examine viral sequences within bulk cellular nucleic acid sample preparations (reviewed by Schmidt [41]), we sought to use virus enrichment as a means to reduce the sequence complexity of the sample while simultaneously providing a cleaner preparation of RNA from sugar beet roots, which can possess compounds inhibitory to many molecular biology procedures. As expected, known sugar beet viruses garnered the greatest number of reads within the samples, and near-complete genome sequences were obtained. Nevertheless, terminal sequences were, in most cases, absent from the assemblies, a general phenomenon characterizing the technique [42]. Those viruses to which the greatest numbers of reads were assigned were predicted to be present in the soil samples used for virus-baiting based on the soil’s cropping history, although the relative number of reads varied between samples submitted and between viruses within a given sample. As the viral RNA was prepared from rhizomania-susceptible sugar beet seedlings harvested in bulk, it was anticipated that this bait would recover a diverse mixture of viruses, and this was born out by our results. By contrast, Sample S6 represents RNA obtained from a single sugar beet plant harvested from a late-season production field and expressing classic rhizomania disease. Viral sequence reads in this sample were dominated by those for BNYVV as compared to bait-plant samples. This is consistent with the fact that Sample S6 was biased, being selected on account of its disease symptoms, as compared to asymptomatic seedlings being bulked from infested soil in the remaining samples.

The results of the present study confirm the power of NGS technologies as applied to RNA-based viral pathogens of sugar beet. Over 98% of the individual genomes of BNYVV, BSBMV, and BBSV were obtained from the read assemblies, with greater than 99% identity in the nucleotide sequence with the closest sequenced relative. A comparison of the assembled sequences for each virus across sample locations within the US indicates a general homogeneity of the sequence. Thus, ~0.13% and 0.2% nt differences between RNA1 and RNA2 sequences were observed, respectively, within the US versus up to 5-fold greater differences between A-type isolates from around the globe (Table 2). An exception to this observation worldwide is nucleotides encoding the “tetrad” of AAs within the p25 protein produced from RNA 3. The nucleotides at this location are considered to be among the most hypervariable within all eukaryotic viruses [25,34,43], a feature observed in our own recent study [17]. Moreover, recent associative [16,17,34] and functional [5,44] evidence suggests that variability in this region may account for the ability for some strains of the virus to circumvent dominant resistance genes in the sugar beet crop. As additional cases exist where a specific tetrad has been observed in both Rz-gene controlled and Rz-gene breaking isolates (e.g., tetrad ACHG [17]), it is possible that other changes in the genome may operate in conjunction with mutations in the tetrad motif or independent of this element in compromising host resistance. The related BSBMV, found only in the US to date, remains a concern as it is not controlled by Rz1 even as it produces only a mild mosaic disease and not the yield losses associated with rhizomania disease [6]. Finally, it was evident that a geographical difference exists for *p25* sequences between isolates in the central states of the US and those existing west of the Rocky Mountain range. This is seen by clustering of the *p25* gene sequence of isolates from Texas, Minnesota, and North Dakota on one branch, separated by additional nodes in the tree from a cluster of isolates obtained from California and Idaho. These specific groupings are also seen for the *p31* sequence, but there are fewer differences between the strains. For both genes, the potential significance of this observation on disease development, vector interaction, or viral fitness remains to be determined. The ability, therefore, to rapidly obtain full genome sequences of BNYVV and other viruses from roots of symptomatic sugar beet will facilitate the detection of other candidate changes conditioning RB in this virus, as well as other viruses or virus variants that may impact the expression of rhizomania disease.

The validity of using RNAseq for the examination of existing, and the discovery of novel, viruses of sugar beet was confirmed through three means in the current study. First, the sequences of BNYVV RNAs 3 and 4 obtained through RNAseq herein were shown to exhibit the closest similarity to archived RNA 3 and RNA 4 sequences from the US that had been obtained in previous studies using standard reverse transcription PCR methods (Figure 2). Several prior investigations utilized sequences of RNAs 3 and 4 for the purposes of categorizing the genetic diversity of BNYVV and ascertaining the origins of the US isolates based on these sequences. We here confirm the grouping proposed by Chiba et al. [25], Schirmer et al. [34], and Zhuo et al. [35], in which US isolates of BNYVV nationwide appear to group with those of the A-types from Italy (Figure 2; Table 2). Although the basis for this apparent relatedness is unknown, it is possible that the virus made its way into US sugar beet production fields through international transit of infested plant material or soil. Transmission of the virus into the US via infected seed can likely be ruled out as no evidence has emerged for the seed-transmission of BNYVV.

Second, the biological validity of the BNYVV sequences obtained was afforded through the construction of clones with demonstrated infectivity based on the consensus sequence from the RNAseq data. The two largest RNAs of the BNYVV genome, RNAs 1 and 2, collectively represent over 70% of the virus genome and encode the replication, packaging, cell–cell movement, and silencing suppression functions of the virus. In contrast to most other biologically-active clones of BNYVV that provide infection through transient genome transcription consequent to *Agrobacterium* infiltration (i.e., “agro-infection”; [44,45,46]), we chose to employ in vitro production of capped RNA transcriptions as the means to produce inoculum for the infection in recipient cells in a manner more consistent with that found in nature. Along with previous reports of others who used NGS data in the construction of clones from which infectious RNA was produced either through in vitro transcription or via agro-infection [47], our study validates this approach in constructing clones for the study of BNYVV.

**Figure 5:**
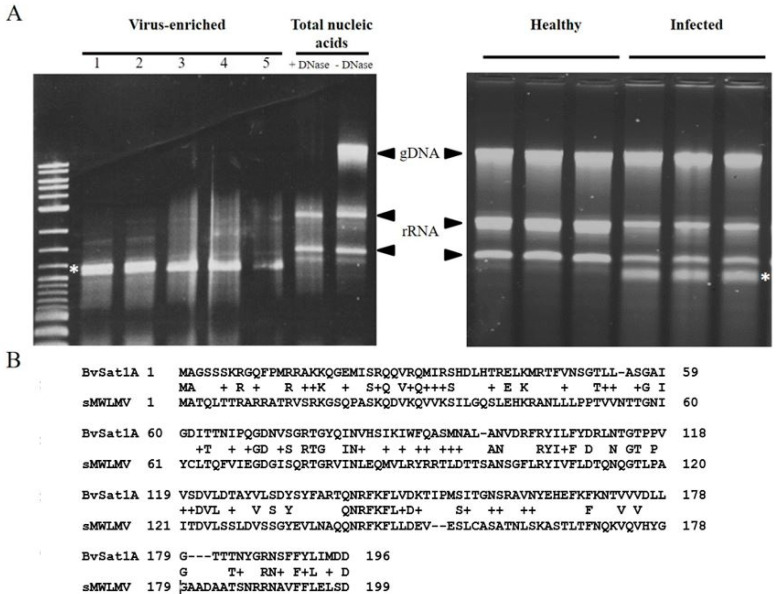
Identification of an abundant satellite virus in sugar beet through virus enrichment and RNAseq. (**A**) Total RNA of healthy sugar beet roots exhibit rRNA and genomic DNA after agarose gel electrophoresis (left panel, -DNase), which can be cleared of DNA (+DNase) prior to RNAseq. Virus enrichment of several infected root samples derived from soils from locations in Minnesota (A, lanes 1–5) results in the persistence of an abundant RNA species of ~1.2 kb present as an apparent doublet band. In the right panel, a high abundance of the satellite virus (*) is evident after agarose gel electrophoresis of total (un-enriched) RNA from healthy and infected plants. (**B**) The single major ORF on the RNA was translated in silico, and the predicted AA sequence (BvSat1A) aligned to that of satellite MWLMV (sMWLMV), the closest relative to the putative novel sugar beet satellite virus in the Genbank database.

Third, the validity of the approach was illustrated by the discovery of a potential novel virus of sugar beet along with a novel satellite virus. The presence of small satellite viruses in sugar beet had not been reported in the USA prior to this study and was unexpected. Analysis of the putative satellite virus genome revealed two variants of a closely-related sequence, both encoding proteins with similarity to themselves and with weak similarity to that encoded by satellite MWLMV, a satellite virus requiring a member of the *Tombusviridae* for its replication [36]. The RNAs differed in size by 157 nt, consistent with the apparent band doublet in a non-denaturing agarose gel (Figure 5). Differences in the size of the two molecules, as predicted from their assembled sequence from that predicted by their electrophoretic migration, may reflect the presence of additional sequences present on those isoforms not captured by RNAseq or conformational aspects of the RNA, resulting in migration anomalies, the possibilities of which are under current investigation.

Further strengthening the validity of this approach in novel virus discovery in sugar beet, the sample in which the satellite virus was present also harbored a previously undocumented variant sequence of TNV-A, possessing a genome organization and gene similarities aligning it with plant Alphanecroviruses within the family *Tombusviridae*. The sequence revealed features suggestive of a recombinant virus with OMMV/OLV-1 and TNV-A as donor parent viruses (Table 3 and Figure 4). The ORF of the putative virus homologous to the replication-associated gene p23 appears to be derived from OMMV, whereas predicted replication protein p52 and movement-associated genes encoding proteins p8 and p6 possess greater similarity to those from OLV-1. At the same time, the predicted CP (p30), the sole CP for members of that sub-genus, appears to be derived from TNV-A in the novel sequence (Table 3 and Figure 4). Since RNAseq data are based on short reads of ~150–200 bp, it has been argued that artifactual contigs might arise during read assembly, potentially providing a false impression of genetic recombination [48]. In the present study, a PCR reaction using primers positioned within the 3′ end of the p52 gene and the 3′ end of the CP gene (Figure 4) yielded a single amplicon, which, when cloned and sequenced, was shown to be homogeneous in sequence and represented the sequence generated by RNAseq. Additionally, within the RNAseq data, no additional “orphaned” ORFs representing other members of the Alphanecroviruses were observed, suggesting that templates of established members of this virus group were absent and could therefore not contribute to the production of artifactual hybrid contigs either through PCR or in silico assembly. Finally, a subsequent genome length amplicon was produced from which RNA was capable of inducing characteristic local lesions on *C. quinoa* (Figure S3). As the sequence of this amplicon matched that obtained through RNAseq within the study, we propose that it represents a new sugar beet-infecting *Alphanecrovirus* within the family *Tombusviridae*, which we propose to be named BvANV-1. Although Liu et al. [49] previously reported TNV from sugar beet in California, no subsequent analysis was conducted to determine the TNV type or its relatedness to other Alphanecroviruses.

A second satellite virus detected in Sample S4 in this study was more closely related to satellite tobacco necrosis virus C, an entity likely associated with helper virus TNV-D or a related Betanecrovirus. Interestingly, Sample S4 harbors BBSV, a well-documented member of that virus subgenus, the US isolate of which was characterized in our laboratory previously [28,32]. Although BBSV is known to have associated satellite RNAs [50], no satellite virus dependent upon BBSV for its replication has, to our knowledge, been reported. The combined compelling results in the present work notwithstanding, for both the novel satellite viruses and candidate helper virus emerging within this study, future investigations will be needed to provide complete sequences and infectious test clones of the genomes. Currently, efforts are underway to test the variant TNV as a helper virus in co-inoculation studies in the presence and absence of the satellite virus.

The NGS approach for detecting known and novel viruses described here and elsewhere provides clear advantages over prior methods of assessment. With reduced bias in the viral genomes targeted, the technique allows for the detection of the presence of unanticipated viruses as compared to more common cloning and sequencing strategies. Moreover, sequence reads for all viruses present are generated simultaneously instead of detecting them within separate, sequential analyses. Standard BLAST alignment can then be employed to detect both known and novel viruses, and more sophisticated applications can detect novel virus agents based on likelihood analysis [51]. Nevertheless, some pitfalls of RNAseq exist due to the short reads produced by the method. Given the existence of virus similarity between members of the same virus family and within family recombinants, one must validate that an assembled sequence represents a true contiguous genome segment through thoughtful primer design and subsequent amplification and cloning of longer sequence segments. As an alternative or complement to RNAseq, one might employ NGS of the like currently offered through PacBio or Oxford Nanopore sequencing platforms that produce long sequence reads from individual molecules as a means to validate sequence contigs generated through RNAseq [52]. Irrespective of the employment of long- or short-read sequencing approaches in initial data acquisition, it appears that 5′-RACE and, to a lesser extent, 3′-RACE will continue to be required in the faithful sequence reproduction of viral RNA genomes and subgenomic transcripts, as 5′- and 3′-terminal structures often are absent or highly underrepresented in sequence reads [42]. Nevertheless, the combination and refinement of NGS technologies have already impacted RNA virology to a great extent in microbial, plant, animal, and human virology, including rapid-response diagnostics and viral genotyping, in recent outbreaks of Ebola virus [53], Zika virus [54], and SARS-CoV2 (COVID-19 infection [55]). As exemplified in the sequencing of multiple genomes of BNYVV and related viruses, and of the discovery of the potentially novel satellite viruses and new Alphanecrovirus reported here, the application of the methods promise to revolutionize detection of known and novel viruses of sugar beet, a crop of global importance.

## Figures and Tables

**Table 1:** RNAseq reads mapped to known sugar beet viruses.

	**Beet Necrotic Yellow Vein Virus (GCF_000854885.1; 15,914 nt) ^a^**													
	**RNA 1** **(NC_003514.1; 6746 nt) ^b^**			**RNA 2** **(NC_003515.1; 4609 nt)**			**RNA 3** **(NC_003516.1; 1774 nt)**			**RNA 4** **(NC_003517.1; 1465 nt)**				
**#**	**Start**	**End**	**Reads Mapped**	**Start**	**End**	**Reads Mapped**	**Start**	**End**	**Reads Mapped**	**Start**	**End**	**Reads Mapped**	**Total Reads Mapped**	**Total Reads**
**S1**	12	6735	46,701	14	4594	82,921	17	1753	36,298	12	1465	37,957	203,877	66,078,080
**S2**	12	6734	121,686	16	4595	240,749	14	1760	71,911	14	1451	123,978	558,325	69,414,580
**S3**	10	6734	97,617	11	4595	235,333	17	1758	52,887	14	1448	93,530	479,367	71,362,360
**S4**	2	6724	15,646	18	4609	21,455	12	1766	12,608	14	1465	5373	55,082	62,246,430
**S5**	6	6727	8369	19	4596	15,041	22	1761	6411	14	1465	2840	32,661	59,595,934
**S6**	5	6744	26,060	16	4609	40,054	1	1744	25,411	14	1465	19,449	110,974	73,945,544
	**Beet Soilborne Mosaic Virus (GCA_002867265.1; 14,744 nt)**													
	**RNA 1** **(JF513082.1; 6679 nt)**			**RNA 2** **(JF513083.1; 4615 nt)**			**RNA 3** **(EU410955.1; 1720 nt)**			**RNA 4** **(FJ424610.2; 1730 nt)**				
**#**	**Start**	**End**	**Reads Mapped**	**Start**	**End**	**Reads Mapped**	**Start**	**End**	**Reads Mapped**	**Start**	**End**	**Reads Mapped**	**Total Reads Mapped**	**Total Reads**
**S1**	12	6671	140,396	16	4597	424,451	1	1720	245,901	1	1714	67,780	878,528	66,078,080
**S2**	12	6671	519,420	16	4595	1,428,890	15	1720	827,273	13	1721	131,926	2,902,509	69,414,580
**S3**	12	6668	153,627	16	4599	677,253	13	1720	217,594	13	1717	162,226	1,210,700	71,362,360
**S4**	7	6676	8696	16	4615	16,635	1	1720	16,177	13	1730	2210	43,718	62,246,430
**S5**	6	6679	83,687	17	4615	157,895	13	1720	133,978	1	1730	56,128	431,688	59,595,934
**S6**	6	6646	738	33	4598	853	16	1702	136	24	1714	24	1751	73,945,544

^a^ Indicates NCBI Accession used as reference and size of genome in nucleotides (nt). Note that 15,914 nt is the size of the BNYVV genome with RNA 5, which is lacking in the United States (USA) strains of the virus; ^b^ Indicates NCBI Accession used as reference and size of genome in nucleotides (nt).

**Table 2:** Similarity between the US BNYVV consensus sequence for RNA 1 and RNA 2 and cognate BNYVV RNAs from outside the USA.

Country of Origin	BNYVV RNA 1					BNYVV RNA 2					
	Accession # ^a^	Identity (%)	Aligned	Mismatch	Gaps	Accession #	Identity (%)	Aligned	Mismatch	Gaps	Type ^b^
Brazil	MH106726.1	99.85	6616	10	0	MH106727.1	99.43	4560	26	0	A
Spain	EU330453.1	99.83	6616	11	0	EU330452.1	99.52	4543	22	0	A
Sweden	EU330450.1	99.80	6616	13	0	EU330451.1	99.44	4533	25	0	A
Yugoslavia	KX665536.1	99.82	6616	12	0	KX665537.1	98.84	4560	32	1	A
Japan	D84410.1	99.37	6616	42	0	D84411.1	98.47	4560	70	0	A
France (Pithiviers)	HM126464.1	99.41	6616	39	0	HM117903.1	98.42	4560	72	0	A(P)
China	KM434313.1	99.43	6616	38	0	KM434314.1	95.66	4563	195	1	B
France	X05147.1	98.46	6616	102	0	X04197.1	95.35	4563	209	1	B

^a^ Indicates NCBI Accession number (#) used as reference; ^b^ The designation of A- versus B*-*type is based on sequence differences within the coat protein gene encoded on RNA 2. Additionally, the presence of RNA 5 contributes to the designation of A(P), originally denoting strains from the Pithiviers region of France.

**Table 3:** Percent identity between the putative novel Necrovirus genome discovered in sugar beet and domains encoded within the genomes of established Alphanecroviruses.

Virus ^a^	Accession # ^b^	5′ UTR ^c^	P23	P52	P8	P6	CP (P30)	3′ UTR
**TNV-A**	GCA_000857065.1	93.22	87.13	94.24	89.04	98.21	93.04	78.04%
**OMMV**	GCF_000858865.1	86.44	96.04	95.01	93.15	98.21	54.13	76.74%
**OLV-1**	GCF_000855965.1	83.05	90.10	95.59	95.89	100.00	49.07	75.80%
**CSNV**	MF125267.1	89.83	86.63	93.67	86.30	98.21	51.65	75.46%
**PoNV**	NC_029900.1	70.00	86.63	89.83	73.97	96.43	50.84	76.05%

^a^ Current members of the *Alphanecrovirus* genus by virtue of primary genome and encoded protein sequence structure; ^b^ Indicates NCBI Accession number (#) used as reference; ^c^ Similarity (%) of the 5′ and 3′ UTR compared at the nucleotide level. Similarity of ORFs for putative proteins P23, P52, P8, P6, and P30 were compared at the amino acid sequence level.

## Supplementary Materials

The following are available online at https://www.mdpi.com/1999-4915/12/6/626/s1, Figure S1: General genome organization of dominant viruses detected through RNAseq applied to sugar beet roots. Figure S2. Sequence analysis of the novel satellite virus genome discovered in sugar beet using RNAseq. Figure S3. Infection of *C. quinoa* with novel *Alphanecrovirus* synthetic RNA based on RNAseq genome data and detection of infecting virus by ELISA. Table S1. Location and form of samples use in this study. Table S2. Primers used in this study.
