# Supplementary material for: RNAseq Analysis of Rhizomania-Infected Sugar Beet Provides the First Genome Sequence of Beet Necrotic Yellow Vein Virus from the USA and Identifies a Novel Alphanecrovirus and Putative Satellite Viruses
Source: Viruses. 2020 Jun 10;12(6):626. doi: 10.3390/v12060626 (PMC7354460; doi:10.3390/v12060626)
Supplement: Supplementary file 1 [file viruses-12-00626-s001.pdf]

Table S1. Location and form of samples use in this study.

Sample number	Sample type	Harvest location	Virus source
S1	Field soil	Wilkin Cty., MN	Bait plants
S2	Field soil	Wilkin Cty., MN	Bait plants
S3	Field soil	Richland Cty., ND	Bait plants
S4	Field soil	Gooding Cty., ID	Bait plants
S5	Field soil	Jerome Cty., ID	Bait plants
S6	Sugar beet root	Renville Cty., MN	Root tissue

Table S2. Primers used in this study.

Primer name	Role	Sequence (5′ – 3′)[a]
MDB-2100	Alphanec P52 For	GTTTGGTATCACACCAGACCTTCAGGAAGCTCTGGAG
MDB-2101	Alphanec P30 Rev	GTTGATAGGTTCAATGAACTCTATCGCGTACTTG
MDB-1867	Satellite coat protein For	CAATACGAAATGGCAGGCTCAT
MDB-1868	Satellite coat protein Rev	ATCAAAGTAACGGACAGTACAA
MDB-2428	RNA 1 fragment 1 For	AAAAGGATCCTAATACGACTCACTATAGAAATTCGATTCTTC
MDB-2376	RNA 1 fragment 1 Rev	GTTAAATCCACTTCAACAGCAAAATTATACTTTGAAGAAG
MDB-2377	RNA 1 fragment 2 For	CTTCTTCAAAGTATAATTTTGCTGTTGAAGTGGATTTAAC
MDB-2378	RNA 1 fragment 2 Rev	TCGGAATAATGAATGATTTTAGTGTCTGGG
MDB-2379	RNA 1 fragment 3 For	CCCAGACACTAAAATCATTCATTATTCCGA
MDB-2380	RNA 1 fragment 3 Rev	TTCCGCTTAAAATATATCTGAGCCGGGGCCACACACATAC
MDB-2381	RNA 1 fragment 4 For	GTATGTGTGTGGCCCCGGCTCAGATATATTTTAAGCGGAA
MDB-2429	RNA 1 fragment 4 Rev	AAAAGGATCCAAGCTTTTTTTTTTTTTTTTTTTTTTTTTTTTTTTTTTTTTTTTTTTTTTTTTTTTTTTTTTTTTATATC
MDB-2460	Alphanec genome For	TAATACGACTCACTATAGAGTATWCATACCAAGWATACS
MDB-2462	Alphanec genome Rev	ATCATATGGGGTGGGGCAAARCCCCTCAATCTG

[a] Red: *Bam*HI restriction site

Orange: T7 promoter sequence

Green: PolyA of 60 nucleotides

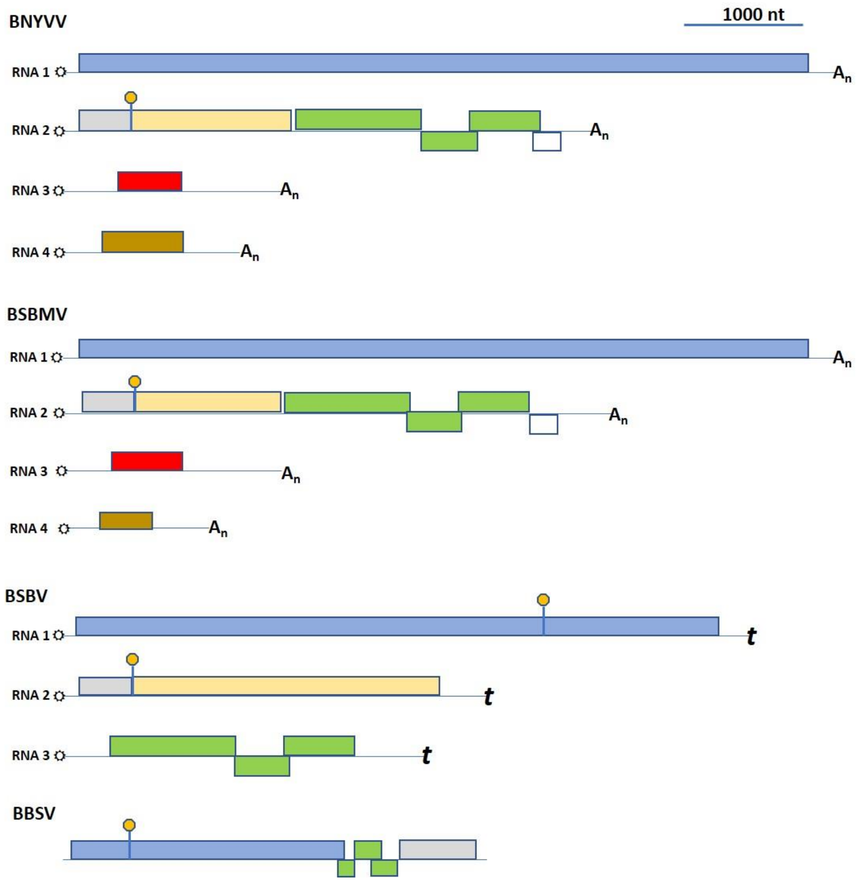

Figure S1. General genome organization of dominant viruses detected through RNAseq applied to sugar beet roots. Lines represent the (+)-sense RNA genomes possessing either no distinguishing terminal features, as in BBSV, or possessing 5′-cap structures (☼) and 3′-polyA tails (**A_n**) or tRNA-like structures (***t***). ORFs (boxes) are known or suspected to encode viral activities for replication (■), encapsidation (■), cell-to-cell movement (■), soilborne vector transmission (■), virus aggressiveness (■), and RNA silencing suppression (□). Suppression of a stop codon (●) to produce read-through proteins is a common theme in the expression of the genomes. Scaling bar = 1000 nucleotides (nt).

A

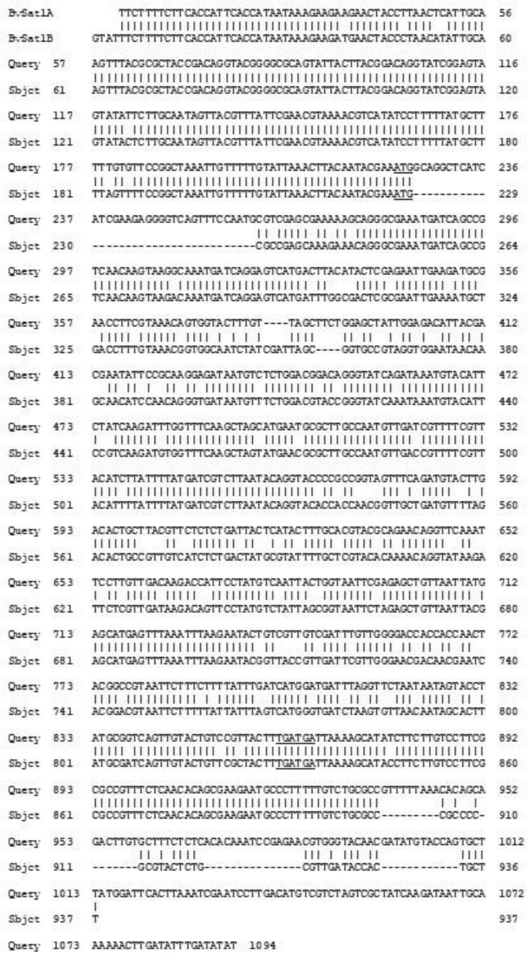

B

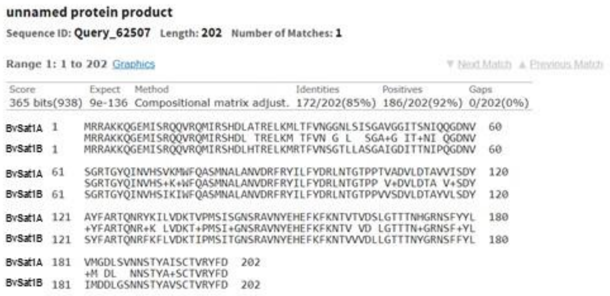

Figure S2. Sequence analysis of the novel satellite virus genome discovered in sugar beet using RNAseq. (**A**) The two isoforms, present in roughly equal proportions in gel electrophoresis and abundance in the RNAseq read pool, are 72% similar at the nt level, with the size difference due to deletions in variant BvSat1B of sequence information present in BvSat1A. (**B**) An in-frame deletion in BvSat1B results in a truncated amino terminus of the predicted coat protein of this variant, lacking the first 12 residues present on the homologous protein of BvSat1A (see Figure 5B). Despite these differences, the level of similarity between the proteins of the two variants as predicted is 85% for that encoded by BvSat1A and BvSat1B.

A

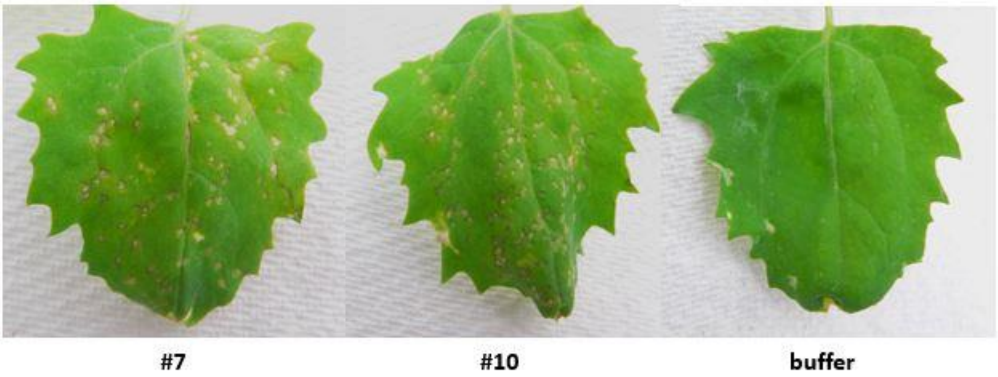

B

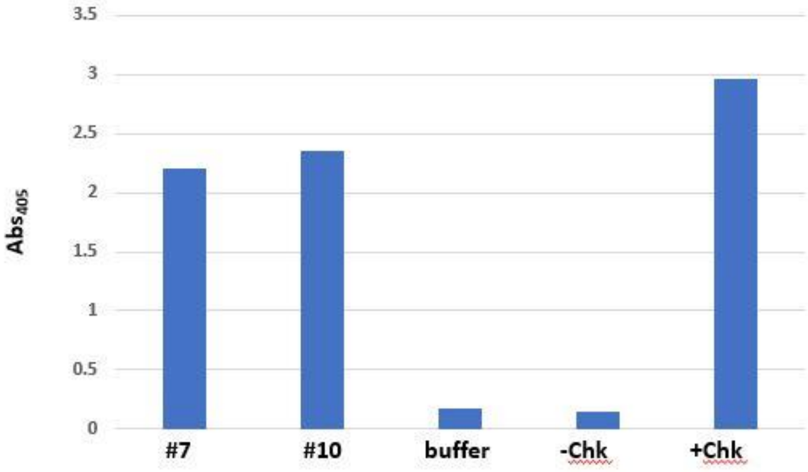

Figure S3. Infection of *C. quinoa* with novel Alphanecrovirus synthetic RNA based on RNAseq genome data and detection of infecting virus by ELISA. (**A**) Expanded leaves of *C. quinoa* plants were inoculated with uncapped RNA transcribed from Clones #7 and #10. Necrotic lesions characteristic of members of the Alphanecroviruses emerged on the inoculated leaves at 5 dpi. (**B**) Infected leaves at 10 dpi produced source extracts for the detection of virus by ELISA using anti-TNV-A antiserum. Reduced levels of antigen detected in leaves inoculated with RNA from Clone-source #7 and #10, relative to that from TNV-A, may be due to differential virus accumulation between these clones and TNV-A (+chk) or to sequence variation between the novel virus and TNV-A in the CP (93% AA sequence similarity). The buffer-inoculated and healthy plant (-chk) controls provide the baseline for determining a positive reaction (positive > 3× baseline = 0.472 OD_{405}).
